# Concurrent and Convergent Validity of a Single, Brief Question for Physical Activity Assessment

**DOI:** 10.3390/ijerph17061989

**Published:** 2020-03-18

**Authors:** Antonio Moreno-Llamas, Jesús García-Mayor, Ernesto De la Cruz-Sánchez

**Affiliations:** San Javier Campus, University of Murcia, 30720 San Javier, Spain; antonio.moreno13@um.es (A.M.-L.); jesus.garcia9@um.es (J.G.-M.)

**Keywords:** physical activity, questionnaires, validation studies, epidemiology

## Abstract

An extensive number of self-reported methods for physical activity (PA) measurement are available, including short and long recall questionnaires ranging from a few to tens of questions. Due to the fact that simple, time-saving methods could be more practical and desirable for use in a busy clinical context, as well as in public health surveys, we evaluated how a single-item question might be a useful and cost-effective method for assessing compliance with PA guidelines. Using multiple receiver operating characteristics (ROC), we assessed the classification performance of a single brief question, employing the short version of the International Physical Activity Questionnaire as criterion instrument, in a total of 55,950 people (30,601 women and 25,349 men). Both those who practice PA almost daily and a few times a week presented an upper threshold (1042.5 metabolic equivalent minutes (MET) minutes/week) to the established compliance PA guidelines (600 MET minutes/week) with high specificity and sensitivity, using a sedentary group as reference. Otherwise, the occasionally physically active group did not reach the minimum (349.5 MET minutes/week) and obtained a poorer classification performance. A single brief question is a pragmatic and alternative method for assessment of compliance with PA guidelines.

## 1. Introduction

The correct assessment of a lifestyle pattern such as physical activity (PA) in clinical and epidemiologic context implies remarkable importance and consequences in order to determine its dose-response relationship with mortality and morbidity [1,2], surveillance of physically inactive population trends across time allowing comparisons [3], or to simply control and classify PA as risk factor into inactive or active behavior. It is found that no practice, and absence of PA, are linked to higher morbidity and all-cause mortality, and lower life quality and quantity, highlighting its influence on non-communicable diseases (NCDs) which is equal to, or even higher than, other lifestyle behaviors as dietary, alcohol use, or tobacco use [1,4,5]. 

Such evaluation could be conducted by means of diverse methodologies and instruments, as either self-reported or direct methods, which involve different and noteworthy applications [2,6]. Examples of direct methods are the use of pedometers, accelerometers, and heart rate monitors; in turn, short- and long-term recall questionnaires, diary notebooks, interviews, and brief questions are encountered among self-reported instruments. Direct measures lessen assessment errors, but some of them are expensive, and almost all mainly require a notorious burden of time (at least one week for most measurement protocols), as well as create logistic issues, such as individual or patient implication [2,6,7]. Contrariwise, self-reported measures are cost-effective tools with high efficiency and capability to easily collect information from large populations or groups; however, these types of instruments present some inherent limitations and biases [2,6]. Thus, the chosen method is underpinned to several factors as assessment purposes, collecting time period, logistics, or effectiveness, among others, and hence, there is not a global gold-standard instrument to evaluate PA pattern.

In certain clinical contexts and large epidemiological studies, the use of self-reported measures could be more feasible. These instruments commonly assess PA by an individual’s recall process in a week, month, or even in more timelines, but they may induce a recall bias, especially during completing large questionnaires, through under- and overestimating PA [2,6]. Besides this, the completion time of widely-used questionnaires is around 10 minutes in short versions, a brief amount of time, but larger for instance, than the average primary care physician consultation time, which is 5 minutes or less in half of the worldwide population, limiting some practical applications [8]. This amount of time could be even larger, whether questionnaires are administered using interviews or by telephone, instead of self-administered forms. Thus, the necessity to employ other instruments and methods which are even shorter, but equally efficient, effective and valid at same time, arise. Few studies have addressed this approach, and mainly focus on older adults with modest sample sizes in their validation procedures [9,10].

The use of a single brief question was therefore proposed as a useful and feasible alternative which, although cannot assess total quantity of PA, at least enables classification into active or inactive in overall population in order to identify as risk factor and/or controlling in epidemiological research, or for screening and exercise prescription in busy primary health care settings. Thereby, we here analyze this assumption through using a brief PA frequency question in comparison to a short-term recall questionnaire as the widely-used International Physical Activity Questionnaire (IPAQ) [11], in a total of 55,950 individuals from 2017 and 2013 Eurobarometers, and the classification of performance into physically active and inactive.

## 2. Methods

### 2.1. Data

Data was retrieved from Eurobarometer survey series. In the present study, Eurobarometer 88.4 [12] and 80.2 [13] from 2017 and 2013, respectively, were selected, as both surveys include IPAQ and a single brief PA frequency question. Each Eurobarometer consists of a cross-sectional survey across the 28 European Union country members, with a representative sample size of approximately 1000 individuals per country, collecting between both surveys a total of 55,950 responders aged 15 and over (30,601 women and 25,349 men).

Sampling was performed by a multi-stage random methodology, drawing several sample points in each country with conditional probability, according to its population size and density among the different stratums and individual units. Age, gender, region, and size of region were used in the iteration process to generate these sample points. Eventually, each participant was randomly selected in each household, and interviews were conducted face-to-face by professional interviewers.

### 2.2. Physical Activity Assessment

Physically active status was considered as compliance with at least one of the World Health Organization (WHO) guidelines: 150 minutes of moderate PA per week, 75 minutes of vigorous PA per week, or any equivalent combination of vigorous and moderate PA [14]. In order to simplify, the WHO guidelines compliance was converted to metabolic equivalent minutes (MET) minutes/week by setting a threshold at 600 MET minutes/week. PA was evaluated by both short-term recall questionnaire employing the short version of IPAQ [11], and a single brief question. 

IPAQ measures PA in a usual week across frequency (number of days) and duration (number of minutes per day) at different intensity levels being vigorous, moderate, and walking. In both Eurobarometers, duration is categorically evaluated by the following answers: “*Never do any vigorous (or moderate) physical activity or Never walk for 10 min at a time”; “30 minor less”; “31 to 60 min”, “61 to 90 min”; “91 to 120 min”; “More than 120 min”*. Categorical duration responses were then transformed using median values for each answer. For example, a duration of 45 minutes was used for the *31 to 60 min* response. For those who reported *Never practice physical activity (or never walk for 10 min)* and *More than 120 min*, a zero value and 135 min were applied, respectively. Subsequently, weekly time at each intensity was computed to Energy Expenditure (EE), expressed as MET per week, that is, the EE compared to resting values [15]. Thus, vigorous, moderate, and walking weekly times were multiplied by 8, 4, and 3.3, respectively, and finally summarized to obtain total EE in a usual week. Further information related to METs for different activities is provided elsewhere [16]. 

Here we study a possible alternative way to rapidly and easily evaluate PA, using the following brief question that was also used in Eurobarometer survey series: *“How often do you exercise, play sport, or engage in other physical activity, such as cycling from one place to another, dancing, gardening, etc.?* Possible answers were practice PA *“Never*”; “*Occasionally”;* “*Few times a week”,* or “*Almost daily”*. 

### 2.3. Data Filtering and Statistical Analyses

Several data filters were applied in order to eliminate potential biases. Firstly, only adults (aged 18 and over) were selected, as PA guidelines are considerably different between adults and adolescents. Secondly, all those responders who reported *Do not know*, or contained missing values either in IPAQ or the brief question, were removed. Furthermore, illogical cases were also eliminated, such as reporting performance of PA 2 days per week and *Never practice physical activity*, or 0 days per week and *61 to 90 min* in the same intensity component (vigorous, moderate and walking) of IPAQ form. This kind of data represents a recall bias and could be capable of negatively affecting statistical analyses, estimations, and interpretation of the results.

Classification of performance was assessed by multiple Receive Operating Characteristic (ROC) curves through MET minutes/week between those who *Never* practice PA, and responders who do *Occasionally, Few times a week*, and *Almost daily*. Therefore, dummy variables were created with *Never* group as reference. Cutoffs were determined by optimizing for both sensitivity (Se) and specificity (Sp). Area under the curve (AUC), positive predicted value (PPV), negative predicted value (NPV), positive likelihood (LH+), and negative likelihood (LH−) were also computed, with 95% confidence interval (95% CI). Rstudio (3.6.1 version) was used to run all statistical analyses and data filtering.

## 3. Results

From a total initial sample size of 55,950 subjects (28,301 in 2017 and 27,919 in 2013), data filtering removed 1103 (1.97%) who were aged under 18; 2,677 (4.78%) who reported *Do not know* as the answer or contained missing values in any of studied variables, and 12,791 (22.86%) illogical cases when reporting IPAQ questions, resulting in a final sample size of 39,379 individuals (70.39%).

Estimated cutoff and corresponding classification performance values are presented in Table 1. For responders who practice PA *Almost daily* and *Few times a week*, the cutoff was set at 1042.5 MET minutes/week, whereas those who practice *Occasionally* had a lower cutoff set at 349.5 MET minutes/week. Moreover, regarding to performance parameters, AUC became greater as frequency grew higher, from *Occasionally* (0.751; 95% CI: 0.743 to 0.759) to *Few times a week* (0.894; 95% CI: 0.891 to 0.898), up to *Almost daily* (0.947; 95% CI: 0.945 to 0.950). Sensitivity, Specificity, and LH+ were also higher among *Almost daily* responders and lower in *Occasionally*; conversely LH– was higher in *Occasionally* (0.401; 95% CI: 0.383 to 0.421) and lower in *Almost daily* (0.127; 95% CI: 0.119 to 0.135). PPV and NPV performance were diverse across groups with no trends. The *Occasionally* group reached a considerably low PPV (0.47; 95% CI: 0.461 to 0.486), in contrast to those who reported practicing PA *Almost daily* (0.862; 95% CI: 0.855 to 0.871) and *Few times a week* (0.904; 95% CI: 0.898 to 0.907), while the later also presented the lowest NPV (0.772; 95% CI: 0.765 to 0.783).

**Figure 1:**
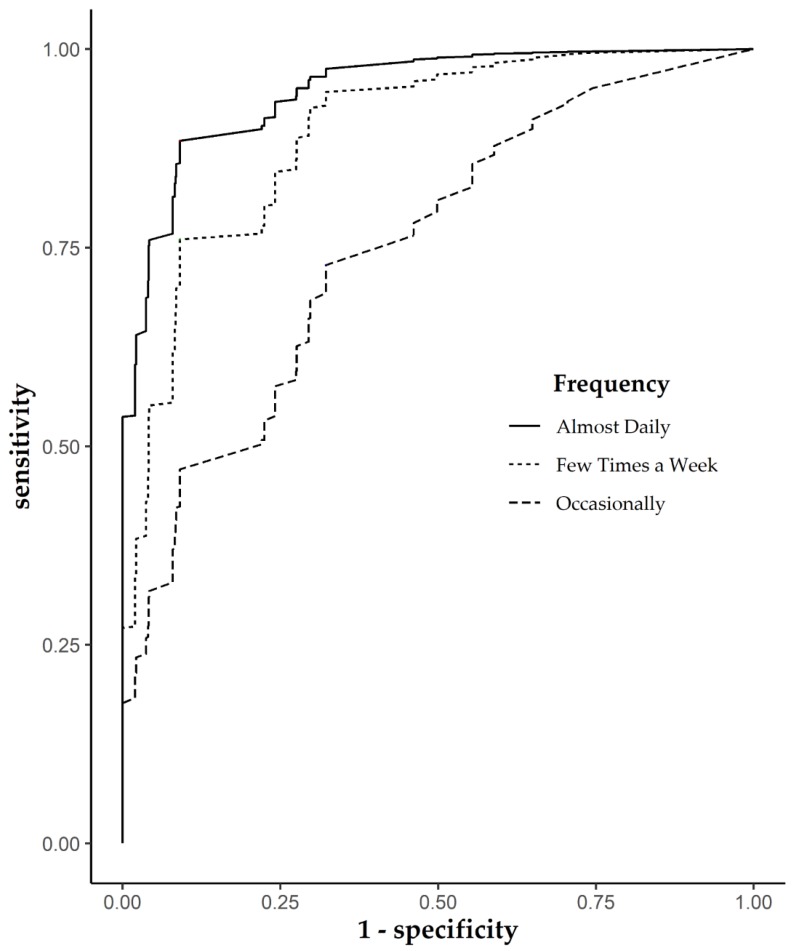
Receive operating characteristic (ROC) curves of different physical activity frequency groups. *Never* group was the reference.

From a visual perspective of classification performance, the *Occasionally* ROC curve differs substantially with respect to the others in a similar way to performance parameters estimated by cutoffs (Figure 1). On the contrary, *Almost daily* and *Few times a week* ROC curves present visually slight differences, and seem to be more similar, compared to the *Occasionally* group.

## 4. Discussion

The ROC results of the present study have shown strong performance in classifying as physically active those who practice PA *Almost daily* or *Few times a week*, with high specificity and sensitivity, especially in the former. The cutoff of 1042.5 MET minutes/week for both groups is placed sufficiently above the threshold of 600 MET minutes/week established compliance with PA guidelines, according to WHO [14]. Nonetheless, classification of performance among those who *Occasionally* practice PA, compared to the sedentary group, was limited, with a cutoff of 349.5 MET minutes/week, lower than the PA compliance threshold. As such, this diminished performance suggests that both groups (*Never* and *Occasionally*) present many similarities; hence, such differentiation results in complex generation of misclassifications. Adding estimated cutoffs to these misclassification problems supports the hypothesis that those who practice PA *Occasionally* could be considered as physically inactive individuals. 

With regard to clinical context, the current self-reported PA assessment tools used in primary care do not present satisfactory properties with respect to validity and reliability [17]. Furthermore, people are more prone to recall PA that involves heavy load or considerable effort (i.e., vigorous activities) than moderate and light activity in daily life [11]. However, the contribution of these vigorous activities represents a small proportion of total time and EE, being more common than light and moderate PA [18]. Recall questionnaires such as IPAQ, which assess PA by splitting into three intensities, may have this type of bias. Therefore, although the exhaustive assessment of PA is also important in order to determine dose-response relationship or simply to control cofounder effects, the use of a single brief question with a global PA assessment could be more suitable, and entails important practical applications, either on large epidemiological surveys or on busy and hasty primary care consultation, allowing for time saved in classification of PA pattern. Other studies have validated similar brief questions and questionnaires [9,10], nonetheless, the employed samples were based on specific population groups, such as older adults, and with limited sample sizes, restricting results’ extrapolation and their applications in other contexts. In contrast, the present study includes a notorious and relevant sample size in a large spectrum of European adults, not heretofore provided in this type of single brief question validation study related to PA assessment.

Besides time-saving applications formerly mentioned, the use of this brief approach to assess PA could also involve strong implications in epidemiological research, either for retrospective purposes or future research. Many cross-sectional survey series present different self-reported instruments, thus, intra- and intercomparability among these surveys would be improved with better PA prevalence surveillance and control, even in already existing surveys, reducing blank periods. Likewise, it should be mentioned that there are a great number of epidemiological studies which measure PA prevalence using this single brief question, and this study would reinforce case control and multiple cross-sectional studies. Lastly, further future research ought to be considered due to the scarce number of this type of validation study of brief questions, especially in different contexts and regions such as individuals with disabilities, or in developing countries. 

In conclusion, the use of a single brief question could be a pragmatic alternative method to assess compliance with PA guidelines of activity, from those who reported practicing PA *Few times a week* or *Almost daily*, to inactive *Never and Occasionally* groups, according to poorer classification performance in the latter. This approach may be suitable in a clinical context as well as in epidemiological and public health research.

## Figures and Tables

**Table 1:** Classification performance parameters with 95% confidence interval (CI) and cutoffs. *Never* group was the reference.

	Almost Daily	Few Times a Week	Occasionally
Cutoff (MET minutes/week)	1042.5	1042.5	349.5
AUC	0.947 (0.945−0.950)	0.894 (0.891−0.898)	0.751 (0.743−0.759)
Se	0.885 (0.877−0.891)	0.760 (0.753−0.767)	0.728 (0.715−0.740)
Sp	0.909 (0.904−0.914)	0.909 (0.904−0.914)	0.678 (0.670−0.686)
PPV	0.862 (0.855−0.871)	0.904 (0.898−0.907)	0.470 (0.461−0.486)
NPV	0.925 (0.920−0.929)	0.772 (0.765−0.783)	0.864 (0.856−0.868)
LH+	9.781 (9.247−10.346)	8.405 (7.944−8.893)	2.260 (2.192−2.331)
LH−	0.127 (0.119−0.135)	0.264 (0.256−0.272)	0.401 (0.383−0.421)

AUC: Area Under the Curve; Se: Sensitivity; Sp: Specificity; PPV: Positive Predicted Value; NPV: Negative Predicted Value; LH+: Positive Likelihood; LH−: Negative Likelihood.

## References

[1] Rhodes R.E., Janssen I., Bredin S.S.D., Warburton D.E.R., Bauman A. (2010). Physical activity: Health impact, prevalence, correlates and interventions. Psychol. Health.

[2] Ainsworth B., Cahalin L., Buman M., Ross R. (2015). The Current State of Physical Activity Assessment Tools. Prog. Cardiovasc. Dis..

[3] Hallal P.C., Andersen L.B., Bull F.C., Guthold R., Haskell W., Ekelund U., Alkandari J.R., Bauman A.E., Blair S.N., Brownson R.C. (2012). Global physical activity levels: Surveillance progress, pitfalls, and prospects. Lancet.

[4] Hallal P.C., Bauman A.E., Heath G.W., Kohl H.W., Lee I.-M., Pratt M. (2012). Physical activity: More of the same is not enough. Lancet.

[5] Kohl H.W., Craig C.L., Lambert E.V., Inoue S., Alkandari J.R., Leetongin G., Kahlmeier S., Andersen L.B., Bauman A.E., Blair S.N. (2012). The pandemic of physical inactivity: Global action for public health. Lancet.

[6] Dowd K.P., Szeklicki R., Minetto M.A., Murphy M.H., Polito A., Ghigo E., van der Ploeg H., Ekelund U., Maciaszek J., Stemplewski R. (2018). A systematic literature review of reviews on techniques for physical activity measurement in adults: A DEDIPAC study. Int. J. Behav. Nutr. Phys. Act..

[7] Corder K., Brage S., Ekelund U. (2007). Accelerometers and pedometers: Methodology and clinical application. Curr. Opin. Clin. Nutr. Metab. Care.

[8] Irving G., Neves A.L., Dambha-Miller H., Oishi A., Tagashira H., Verho A., Holden J. (2017). International variations in primary care physician consultation time: A systematic review of 67 countries. BMJ Open.

[9] Taylor-Piliae R.E., Norton L.C., Haskell W.L., Mahbouda M.H., Fair J.M., Iribarren C., Hlatky M.A., Go A.S., Fortmann S.P. (2006). Validation of a new brief physical activity survey among men and women aged 60-69 years. Am. J. Epidemiol..

[10] Martínez-Gómez D., Guallar-Castillón P., Higueras-Fresnillo S., Rodríguez-Artalejo F. (2017). Concurrent Validity of the Historical Leisure-time Physical Activity Question of the Spanish National Health Survey in Older Adults. Rev. Española Cardiol..

[11] Craig C.L., Marshall A.L., Sjöström M., Bauman A.E., Booth M.L., Ainsworth B.E., Pratt M., Ekelund U., Yngve A., Sallis J.F. (2003). International physical activity questionnaire: 12-Country reliability and validity. Med. Sci. Sports Exerc..

[12] European Commission Eurobarometer 88.4 (2017). TNS Opinion, Brussels.

[13] European Commission Eurobarometer 80.2 (2013). TNS Opinion, Brussels.

[14] (2010). World Health Organization Global Recommendations on Physical Activity for Health.

[15] Ainsworth B.E., Haskell W.L., Herrmann S.D., Meckes N., Bassett D.R., Tudor-Locke C., Greer J.L., Vezina J., Whitt-Glover M.C., Leon A.S. (2011). 2011 compendium of physical activities: A second update of codes and MET values. Med. Sci. Sports Exerc..

[16] Ainsworth B.E., Haskell W.L., Whitt M.C., Irwin M.L., Swartz A.M., Strath S.J., O’brien W.L., Bassett D.R., Schmitz K.H., Emplaincourt P.O. (2000). Compendium of Physical Activities: An update of activity codes and MET intensities. Med. Sci. Sport. Exerc..

[17] Smitha T.O., McKenna M.C., Salter C., Hardeman W., Richardson K., Hillsdon M., Hughes C.A., Steel N., Jones A.P. (2017). A systematic review of the physical activity assessment tools used in primary care. Fam. Pract..

[18] Westerterp K.R. (2001). Pattern and intensity of physical activity. Nature.

